# Foundation model-driven distributed learning for enhanced retinal age prediction

**DOI:** 10.1093/jamia/ocae220

**Published:** 2024-09-03

**Authors:** Christopher Nielsen, Raissa Souza, Matthias Wilms, Nils D Forkert

**Affiliations:** Department of Radiology, University of Calgary, Calgary, AB T2N 4N1, Canada; Biomedical Engineering Graduate Program, University of Calgary, Calgary, AB T2N 4N1, Canada; Department of Radiology, University of Calgary, Calgary, AB T2N 4N1, Canada; Biomedical Engineering Graduate Program, University of Calgary, Calgary, AB T2N 4N1, Canada; Hotchkiss Brain Institute, University of Calgary, Calgary, AB T2N 4N1, Canada; Department of Radiology, University of Calgary, Calgary, AB T2N 4N1, Canada; Hotchkiss Brain Institute, University of Calgary, Calgary, AB T2N 4N1, Canada; Alberta Children’s Hospital Research Institute, University of Calgary, Calgary, AB T2N 4N1, Canada; Department of Pediatrics, University of Calgary, Calgary, AB T2N 4N1, Canada; Department of Community Health Sciences, University of Calgary, Calgary, AB T2N 4N1, Canada; Department of Radiology, University of Calgary, Calgary, AB T2N 4N1, Canada; Hotchkiss Brain Institute, University of Calgary, Calgary, AB T2N 4N1, Canada; Alberta Children’s Hospital Research Institute, University of Calgary, Calgary, AB T2N 4N1, Canada; Department of Clinical Neurosciences, University of Calgary, Calgary, AB T2N 4N1, Canada

**Keywords:** retinal age prediction, distributed learning, retinal age gap, foundation models, machine learning

## Abstract

**Objectives:**

The retinal age gap (RAG) is emerging as a potential biomarker for various diseases of the human body, yet its utility depends on machine learning models capable of accurately predicting biological retinal age from fundus images. However, training generalizable models is hindered by potential shortages of diverse training data. To overcome these obstacles, this work develops a novel and computationally efficient distributed learning framework for retinal age prediction.

**Materials and Methods:**

The proposed framework employs a memory-efficient 8-bit quantized version of RETFound, a cutting-edge foundation model for retinal image analysis, to extract features from fundus images. These features are then used to train an efficient linear regression head model for predicting retinal age. The framework explores federated learning (FL) as well as traveling model (TM) approaches for distributed training of the linear regression head. To evaluate this framework, we simulate a client network using fundus image data from the UK Biobank. Additionally, data from patients with type 1 diabetes from the UK Biobank and the Brazilian Multilabel Ophthalmological Dataset (BRSET) were utilized to explore the clinical utility of the developed methods.

**Results:**

Our findings reveal that the developed distributed learning framework achieves retinal age prediction performance on par with centralized methods, with FL and TM providing similar performance (mean absolute error of 3.57 ± 0.18 years for centralized learning, 3.60 ± 0.16 years for TM, and 3.63 ± 0.19 years for FL). Notably, the TM was found to converge with fewer local updates than FL. Moreover, patients with type 1 diabetes exhibited significantly higher RAG values than healthy controls in all models, for both the UK Biobank and BRSET datasets (*P* < .001).

**Discussion:**

The high computational and memory efficiency of the developed distributed learning framework makes it well suited for resource-constrained environments.

**Conclusion:**

The capacity of this framework to integrate data from underrepresented populations for training of retinal age prediction models could significantly enhance the accessibility of the RAG as an important disease biomarker.

## Introduction

The retinal age gap (RAG) has recently gained attention as a promising disease biomarker, showing clinically relevant correlations with several diseases, including metabolic syndrome, neurodegenerative conditions, and kidney failure.^1–5^ The RAG measures the difference between an individual's chronological age and their retina's biological age, determined using a machine learning (ML) model trained using retinal imaging data from healthy subjects. Importantly, the RAG can be acquired using non-invasive, readily available, and affordable fundus imaging technology. This imaging technology is widely used in eye clinics globally and is adaptable to mobile devices for accessible use in homes and rural settings.^6^^,^^7^

The effectiveness of the RAG as a biomarker relies on the accuracy of ML models used for retinal age prediction.^2^ However, a significant challenge faced when developing ML models, especially impacting rural and resource-constrained regions, is the potential lack of training data from these underrepresented groups.^8^^,^^9^ This lack of data can lead to selection bias,^10^ compromising the models' performance and generalizability.^11^ Hence, it is essential that training datasets appropriately represent the demographic characteristics of the target populations.

**Figure 1.**
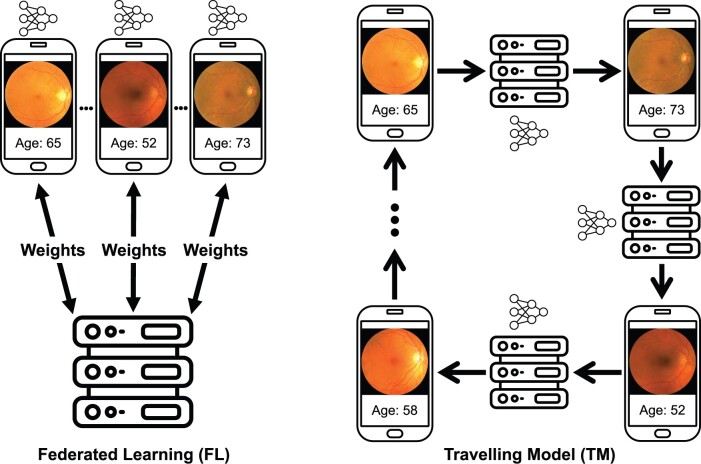
Distributed learning techniques performed over a network of client mobile devices. Federated learning (FL) is shown on the left and traveling model (TM) on the right.

Distributed learning, an emerging ML training paradigm, has potential to help expand the training data volume and diversity without explicit data sharing.^12^ This method enables training across client devices without transferring sensitive data to a central location. Federated learning (FL), the most widely adopted form of distributed learning, involves sending model parameters from a central server to individual clients for local training.^13^ Subsequently, the parameter updates are sent back to the central server and aggregated to refine the global model. This process is repeated until the global model meets a predetermined criterion for convergence. An alternative strategy, the traveling model (TM) approach, trains a single global model by sending it to each client sequentially for local training, eliminating the need for centralized model aggregation.^14^^,^^15^ Importantly, Souza et al. reported that TM may perform better in scenarios with smaller datasets.^16^Figure 1 illustrates these methods across a network of mobile devices.

Several distributed learning methods have been developed for ocular disease prediction.^17^ For instance, Gholami et al. showcased the application of FL for training vision transformer (ViT) models to detect age-related macular degeneration.^18^ Furthermore, Yan et al. illustrated the use of FL in training ViT models for detecting multiple eye diseases, including diabetic retinopathy and retinal vein occlusion.^19^ However, these strategies necessitate intensive computational finetuning and full data transfer of large ViT models during each local client update. These requirements could restrict usability for regions with limited resources. Additionally, distributed learning has not yet been explored for the retinal age prediction task.

RETFound, a state-of-the-art ViT foundation model developed for analyzing retinal fundus images, has proven to be highly effective across a broad spectrum of ophthalmic predictive tasks.^20^ Pretrained on more than 0.9 million fundus images through self-supervised learning, RETFound is capable of extracting informative lower-dimensional feature vectors from fundus images that could be used to build rather simple machine learning models for downstream tasks. Furthermore, the feasibility of using of ViT models for inference on mobile devices such as smartphones has been demonstrated in several recent studies.^21–23^

Therefore, this work introduces a novel distributed learning framework for retinal age prediction by utilizing features from retinal fundus images extracted by a pre-trained, non-finetuned RETFound encoder. These features are then used by a trainable compact linear regression head (LRH) model for predicting retinal age. The key contributions of this work can be summarized as follows: (1) To the best of our knowledge, this is the first work exploring the use of distributed learning for retinal age prediction. (2) We developed an efficient distributed learning framework that utilizes retinal fundus image features extracted by an 8-bit quantized RETFound encoder to train a computationally efficient but powerful LRH model for retinal age prediction. (3) We investigate how FL and TM strategies compare in performance to traditional centralized learning methods with varying amounts of training data. (4) We evaluate the clinical utility of the trained models by determining the average RAG for patients with type 1 diabetes and comparing these values to those of healthy controls.

## Methods

### Data sources

This work primarily utilized data from the UK Biobank, a crucial biomedical research resource.^24^ Established to collect genetic and health data from over 500 000 UK individuals, the UK Biobank supports research into the prevention, diagnosis, and treatment of various diseases. From 2006 to 2010, initial data collection was carried out across 22 centers in the United Kingdom, targeting individuals aged 40-69. Notably, the UK Biobank has gathered retinal fundus images from over 80 000 subjects, using the non-mydriatic Topcon 3D OCT-1000 Mark II scanner to ensure a 45-degree retinal view focusing on the optic disc and macula. The UK Biobank also includes 10th Revision of the International Statistical Classification of Diseases and Related Health Problems (ICD-10) codes for each patient.

Additionally, the Brazilian Multilabel Ophthalmological Dataset (BRSET) served as an external validation source to assess the clinical relevance of the framework.^25^^,^^26^ This dataset includes 16 266 images from 8524 participants, collected between 2010 and 2020, with each participant providing just one macula-centered paired exam. The retinal photos were taken with a Nikon NF505 and a Canon CR-2 fundus camera. Furthermore, the BRSET dataset contains information on comorbidities impacting each patient.

### Dataset construction and preprocessing

In this work, a total of 177 354 fundus images (left and right eyes) were gathered from 86 522 subjects within the UK Biobank dataset. To align with the input dimensions required by the RETFound model, these images underwent preprocessing. This involved centering the retina within the image frame and resizing the images from the initial 2048 × 1536 pixels down to 224 × 224 pixels.^20^ Additionally, the deep learning methodology developed by Fu et al. was used to filter out images with poor acquisition quality.^27^ This led to the selection of 97 611 high-quality images from 56 258 subjects. To determine the subset of healthy subjects for training the retinal age prediction models, the criteria established by Zhu et al. were followed.^2^ This involved considering subjects as healthy if they had no self-reported medical conditions prior to the collection of their imaging data. Furthermore, fundus images from both eyes of these healthy subjects were used, resulting in a dataset comprising 16 630 images from 8315 unique healthy subjects. Table 1 provides detailed demographic information for these subjects. These images were acquired from 8 different medical centers across the United Kingdom. To assess the generalizability of the trained models, data from 3 centers (1482 healthy subjects) in England and Wales were used exclusively for external testing, while data from the remaining 5 centers (6833 healthy subjects) were utilized for training and internal validation. Additionally, ICD-10 clinical codes (see Table S3) were utilized to identify 238 images from 119 participants (both left and right eyes) within the UK Biobank who, having passed retinal image quality control, were diagnosed with type 1 diabetes before the imaging took place.

**Table 1.** Demographic information of the 8315 healthy subjects in UK Biobank utilized for training and evaluating the distributed learning framework.

	N	Mean	Std	Min	Q1	Q2	Q3	Max
Age (years)	8315	52.32	7.98	40	45	52	59	74
Diastolic blood pressure (mmHg)	8272	80.19	9.65	45	73.5	79.5	86.5	118.5
Systolic blood pressure (mmHg)	8272	131.97	16.87	84.5	120	130	142.5	226.5
HDL cholesterol (mmol/L)	7184	1.53	0.39	0.53	1.25	1.47	1.76	3.41
Triglycerides (mmol/L)	7587	1.54	0.93	0.25	0.93	1.27	1.84	11.01
Glucose (mmol/L)	7174	5	0.63	2.91	4.67	4.95	5.26	19.8
Weight (kg)	8285	75.4	14.68	40.3	64.5	74	84.8	166.6
Body mass index (kg/m^2^)	8281	26.11	4.08	15.16	23.29	25.66	28.36	51.34
Arterial stiffness index	8207	8.91	4.69	2.04	6.76	8.42	10.53	350
Left eye logMAR	8271	0	0.2	−0.48	−0.12	−0.06	0.06	1.35
Right eye logMAR	8283	0	0.2	−0.44	−0.12	−0.06	0.06	1.35
Alcohol intake	8292	Never: 513, Special occasions only: 872, One to 3 times a month: 959, Once or twice a week: 2246, 3 or 4 times a week: 2068, Daily or almost daily: 1624, Prefer not to answer: 10						
Smoking status	8292	Never smoked: 5027, Previous smoker: 2420, Current smoker: 821, Prefer not to answer: 24						
Sex	8315	Female: 4526, Male: 3789						
Ethnicity	8292	White: 7604, Mixed background: 75, Black: 203, Asian: 216, Other: 194						

N, number of subjects; Std, standard deviation; Min, minimum observed value; Q1, quartile 1; Q2, quartile 2; Q3, quartile 3; Max, maximum observed value; logMAR, logarithm of the minimum angle of resolution.

The same procedures used for preprocessing of the UK Biobank images were also used for the BRSET dataset, which resulted in a total of 958 images from 479 healthy participants (both left and right eyes). Table 2 provides demographic information for these subjects. In the BRSET dataset, a participant was deemed healthy if they exhibited no eye diseases or comorbidities. Furthermore, 428 images from 214 participants (both left and right eyes) in the BRSET dataset had a prior reported comorbidity of type 1 diabetes.

**Table 2.** Demographic information of the 479 healthy subjects in BRSET dataset utilized for external testing.

	N	Mean	Std	Min	Q1	Q2	Q3	Max
Age (years)	479	46.97	17.46	6	33	49	60	91
Sex	479	Female: 326, Male: 153						

N, number of subjects; Std, standard deviation; Min, minimum observed value; Q1, quartile 1; Q2, quartile 2; Q3, quartile 3; Max, maximum observed value.

### Simulated distributed data

To evaluate the performance of the distributed learning framework, 10 Monte-Carlo cross-validation iterations were performed. In each Monte-Carlo iteration, datasets composed of healthy subjects were constructed for training and internal validation using random sampling. Specifically, 1200 healthy subjects were sampled without replacement for internal validation. Additionally, to assess how changes in the training set size affect the framework's effectiveness, training sets comprising 150, 300, 600, 1200, and 2400 healthy subjects were also sampled. To avoid statistical bias, data from each subject were meticulously divided to ensure that images from the same subject did not overlap between the training, internal validation, and external testing datasets, thus preventing cross-contamination. After the models were trained, they were evaluated on the internal validation and external testing datasets to assess model generalizability.

In this work, we specifically investigate the distributed learning setup where each simulated client in the network possessed data from only one healthy individual. This scenario could mirror a real-world situation where individual clients correspond to the personal mobile devices of unique healthy subjects. To emulate the distributed client network, every healthy subject within the training, internal validation, and external testing datasets was allocated to a distinct simulated client within the distributed learning framework. Each simulated client held precisely 2 images from their respective healthy subject, one from the left eye and one from the right eye. Furthermore, for the development of a centralized learning baseline model, data from the simulated clients were consolidated to create centralized datasets for training, internal validation, and external testing.

### RETFound-based feature vector extraction

In this work, feature vectors for each fundus image are computed by processing the images through a forward pass through the RETFound encoder model.^20^ To adapt the feature vector extraction process for mobile devices with restricted memory capabilities, model quantization was implemented using the PyTorch Quantized Neural Networks PACKage (QNNPACK).^28^ This reduced the numerical precision of the RETFound encoder parameters from the original 32-bit precision to 8-bit quantized values, notably reducing the memory requirements for storing the encoder parameters by roughly 4-fold, from approximately 1.2 GB to around 300 MB. To investigate the effects of quantization on model performance, experiments were conducted with both 8-bit quantized and uncompressed RETFound feature vectors during training with centralized data.

### Distributed learning workflow

**Figure 2.**
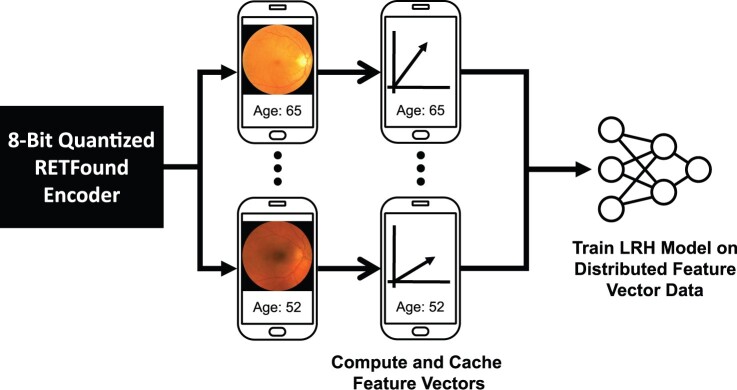
Workflow for training retinal age prediction models on distributed data. An 8-bit quantized RETFound encoder is first sent to or directly installed on each of the client devices, which contain the client’s private fundus images. The 8-bit quantized RETFound encoder is then used to extract feature vectors for each fundus image. These feature vectors are cached locally on the device and used during distributed learning to train the linear regression head (LRH) model for retinal age prediction.

Figure 2 presents a diagram of the proposed distributed learning system. In the initial stage, the 8-bit quantized RETFound encoder model is dispatched to each client in the distributed learning network. The primary function of the encoder model is to generate compact feature vectors from fundus images, which are stored locally on the client devices. Importantly, this step of extracting image features is a one-time requirement at the start of the training regime. Afterwards, there is no further need for either the original fundus images or the 8-bit quantized RETFound encoder model as the training of the final global LRH model is performed exclusively using these derived feature vectors. This paves the way for the actual training of the global LRH model, which is performed using either FL or TM methodologies.

During each round of FL training, the latest version of the global LRH model is sent to a group of 32 simulated clients, randomly sampled from the training set at the start of the round, for local training. Following the completion of local training, the updated global LRH model parameters are sent back from each simulated client to the central server and combined by averaging them, a method known as federated averaging (FedAvg).^29^

Conversely, the TM training approach adopts a sequential methodology for updating the global LRH model parameters. This method involves sequentially dispatching the global LRH model to individual simulated clients. After local training is completed for one simulated client, the model parameters are transferred to the next simulated client, either directly or through the central server. This process is repeated until the global LRH model reaches the last client in the network, finishing the training cycle. Furthermore, the order in which clients are visited is randomized each cycle to protect against bias and catastrophic forgetting.^30^^,^^31^ For both the FL and TM models, upon completion of each training round/cycle, convergence is assessed by distributing the global LRH model to simulated clients using the internal UK Biobank validation set. This is done to evaluate performance on local validation data and determine if the early stopping criteria have been met. After training is completed, the global LRH model is dispatched to simulated clients within the external test sets for performance evaluation on their test data not used for training to evaluate the trained model's generalizability.

### LRH model training

The evaluation of the proposed framework's performance was conducted using 3 distinct training setups: (1) distributed learning using FL, (2) distributed learning using TM, and (3) centralized learning as a benchmark for performance comparison. Each setup utilized the same LRH model architecture, which is a linear regression model comprising 1025 trainable parameters (1024 weights plus one bias term). The goal of the LRH model training was to reduce the L2 loss between the actual chronological age and the predicted age. This optimization was carried out using the Adam optimizer, with a learning rate of ${5\times 10}^{-3}$ and weight decay of ${1\times 10}^{-4}$. In the distributed learning scenarios, local training on each client device was conducted over 200 rounds/cycles, incorporating an early stopping mechanism to halt training if no validation performance improvement was observed after 10 rounds/cycles. Similarly, for centralized learning, the training spanned 200 epochs with an early stopping criterion triggered after 10 epochs without gain in performance.

In order to investigate the performance of the proposed simple LRH model during centralized learning compared to more complex head models, 3 multilayer perceptron (MLP) models were developed using 8-bit quantized RETFound feature vectors. These MLP models included configurations with 2, 3, and 4 layers, with each layer containing 100 hidden units. The training approach for these models mirrored that of the LRH models.

### Statistical analysis and performance evaluation

Retinal age prediction performance was evaluated using the mean absolute error (MAE) measured between chronological age and the predicted age. The calculation of the floating-point operations (FLOPs) needed for each model architecture was carried out using the calflops Python library.^32^ To determine the minimum computational demands of the simulated client network within the distributed framework, we computed the total FLOPs necessary for all gradient calculations and optimizer updates during training. Similarly, we estimated the minimum amount of data transmitted over the communication network by determining the number of times when model parameters were shared between simulated clients and the server throughout the training process.

To assess the statistical significance of performance differences between 2 models, we utilized 2-sided t-tests. Additionally, for comparing multiple models, 1-way analysis of variance (ANOVA) was employed. Considering the multiple comparisons involved in testing performance differences across 5 distinct dataset group sizes and the 3 models being compared, the Bonferroni correction method was applied. This approach adjusted the significance threshold by setting $\alpha =\frac{0.05}{15}$. A *P*-value falling below this adjusted α threshold was considered a significant indicator of performance variation between the models.

To evaluate the clinical utility of the developed framework, models trained with data from 2400 UK Biobank participants were used to calculate the RAG values for both healthy individuals and those with type 1 diabetes within the external UK Biobank and BRSET datasets. RAG values were calculated as the difference between the predicted biological age and the chronological age. Subsequently, domain adaptation techniques, including weighted bootstrap resampling, were applied to adjust for the differences between the age distribution of the training set and that of the target groups.^33^ Within this context, healthy participants are expected to have an average RAG value of zero. One-tailed nonparametric bootstrap hypothesis testing was conducted to determine if the average RAG scores for participants with type 1 diabetes were significantly different from those for healthy controls. A Bonferroni correction was applied to the significance threshold, setting $\alpha =\frac{0.05}{6}$, to account for multiple comparisons across the 3 model types (centralized, TM, and FL) and 2 datasets (UK Biobank and BRSET).

## Results

### Baseline performance for centralized data with and without quantization

**Figure 3.**
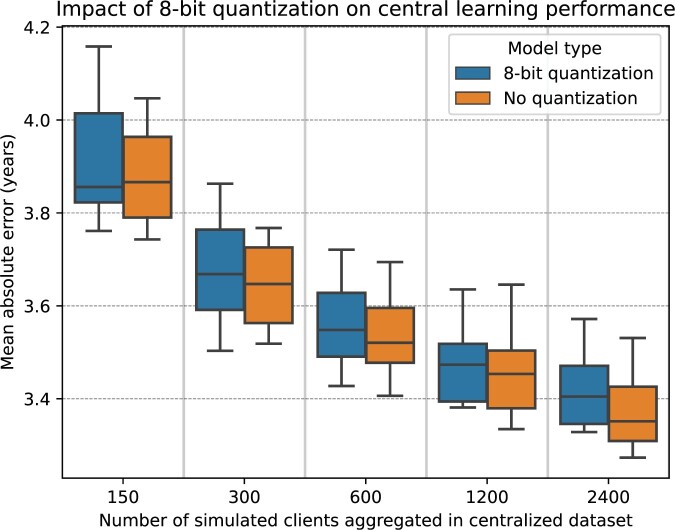
Box plots of the performance comparison, evaluated on the internal validation dataset, using mean absolute error between RETFound-based linear regression models trained on centralized data with 8-bit quantization (depicted by blue boxes) and those trained without quantization (depicted by orange boxes).

Figure 3 showcases the efficiency of the LRH model, evaluated on the internal validation dataset, under centralized training conditions, employing both 8-bit quantized and uncompressed RETFound feature vectors. As the number of images in the training dataset grew, the performance of both types of models improved. For instance, with aggregated data from 2400 simulated clients, the MAE for the models using 8-bit quantization was 3.42 ± 0.09 years, while the MAE for the uncompressed models was 3.37 ± 0.08 years. Moreover, although the uncompressed RETFound feature vectors tended to perform slightly better, the statistical analysis revealed no statistically significant differences in the performance outcomes between the 8-bit quantized and the unquantized models (*P* = .45).

Using MLP models trained with aggregated data from 2400 simulated clients, the MAE for models employing 8-bit quantization was recorded as follows: 3.44 ± 0.11 years for the 2-layer MLP, 3.41 ± 0.07 years for the 3-layer MLP, and 3.42 ± 0.10 years for the 4-layer MLP. The analysis showed no statistically significant differences in performance outcomes between the LRH models and the larger MLP models (*P* = .62).

### Distributed learning performance

**Figure 4.**
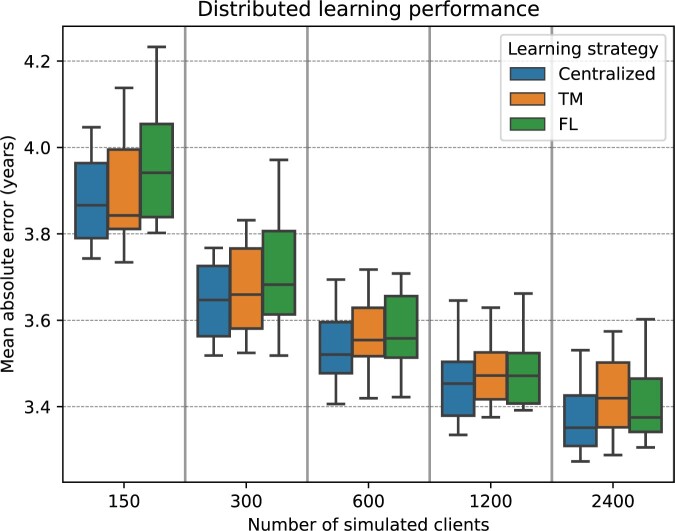
Box plots demonstrating the performance of distributed learning methods versus traditional centralized learning for predicting retinal age based on the mean absolute error, evaluated on the internal validation dataset.

Figure 4 showcases the efficacy of distributed learning techniques, evaluated on the internal validation dataset, compared to conventional centralized learning in the context of retinal age prediction, focusing on the MAE metric. As the number of simulated clients in the training dataset increased, all types of models showed improved performance, as evidenced by a decrease in MAE. For instance, using training data from 2400 participants, centralized learning recorded an MAE of 3.37 ± 0.08 years, while the TM and FL strategies achieved MAEs of 3.42 ± 0.09 years and 3.41 ± 0.10 years, respectively. Interestingly, the results demonstrate that there were no statistically significant differences in performance between centralized and distributed learning models (*P* = .46). Similarly, no significant performance differences were observed between the TM strategy and the FL approach (*P* = .68). The average MAE computed across all training dataset sizes was 3.57 ± 0.18 years for centralized learning, 3.60 ± 0.16 years for the TM strategy, and 3.63 ± 0.19 years for FL.

To assess the generalizability of the trained models, the MAE was calculated for the external testing datasets. Detailed results can be found in Table S1. For each type of model, the MAEs computed for the UK Biobank external testing dataset were consistently slightly higher than those on the internal validation dataset. For instance, the TM strategy yielded an MAE of 3.42 ± 0.09 years for the internal validation dataset compared to 3.60 ± 0.14 years for the UK Biobank external testing dataset when trained using data from 2400 clients. Similarly, the FL strategy led to an MAE of 3.41 ± 0.10 years on the internal validation dataset versus 3.56 ± 0.10 years on the UK Biobank external testing dataset. Nevertheless, the average MAE increase between the internal validation and external testing datasets across all trained models was just 4.44%, suggesting robust generalizability of the models. Furthermore, the findings show that there were no statistically significant differences in performance between centralized and distributed learning models on the external testing dataset (*P* = .20). Likewise, no significant differences in performance were noted between the TM strategy and the FL approach on the external testing dataset (*P* = .60).

Additionally, when models trained on data from 2400 UK Biobank participants were used to estimate retinal age in the healthy participants from the external BRSET dataset, the MAE for the model trained using centralized learning was 4.20 ± 0.11 years. For the TM strategy, the MAE was 4.23 ± 0.14 years, and for the FL strategy, the MAE was 4.24 ± 0.13 years. Notably, there were no significant differences in performance among the centralized, TM, and FL methods (*P* = .51).

### Distributed learning computational and data transfer efficiency

At the start of the training process, the 8-bit quantized RETFound encoder is already pre-loaded or dispatched to every simulated client device and executed a single time to extract feature vectors from locally stored fundus images. This initial task necessitates around 119.3 GFLOPs to process the feature vectors for each image and involves a data transfer of 303.3 MB to send the encoder to the simulated clients, if not already available. In following stages of distributed learning, the LRH model can be trained in a highly efficient manner, requiring merely 2.0 kFLOPs for inference and 15.3 kFLOPs for training updates. Furthermore, the LRH model is composed of 1025 trainable parameters, which, combined with the optimizer's state parameters, use about 15.4 KBs of memory. The LRH model provides superior efficiency relative to training larger MLP models. Specifically, the 2-layer MLP necessitated 615.3 kFLOPs for inference and 2.8 MFLOPs for training updates, the 3-layer MLP used 675.6 kFLOPs for inference and 3.0 MFLOPs for training updates, while the 4-layer MLP required 735.9 kFLOPs for inference and 3.3 MFLOPs for training updates. Additionally, this approach offers a significant efficiency boost over fully finetuning the RETFound model, which requires 119.3 GFLOPs per image for inference and 360.8 GFLOPs for training updates.

**Figure 5.**
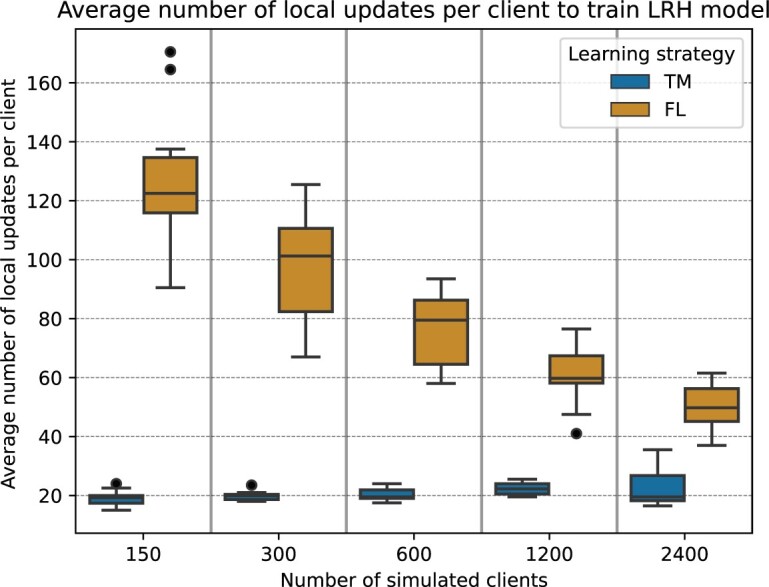
Box plots showing the average number of simulated client local training iterations required to train the linear regression head (LRH) model.

Figure 5 presents the mean number of local training iterations needed per simulated client to train the LRH model. Notably, the TM learning approach outperforms FL in terms of computational and data transfer efficiency. However, the TM strategy shows a slight decline in computational efficiency with an increase in number of simulated clients. For instance, under the TM strategy, with 150 clients, the average per-client computational and data transfer was 436.48 ± 82.99 kFLOPs and 0.22 ± 0.04 MBs, respectively, while for 2400 clients, these figures increased to 540.99 ± 194.51 kFLOPs and 0.27 ± 0.10 MBs. Additional details regarding the average computational and data transfer demands per client for training the LRH models are detailed in Table S2.

### RAG value analysis

To evaluate the clinical utility, average RAG values were computed for both healthy participants and patients with type 1 diabetes, using data from the external UK Biobank and BRSET datasets. The detailed findings are available in Table S4. Consistently, across all models (centralized learning, TM, and FL), and both external datasets, the RAG values for patients with type 1 diabetes were significantly higher than that for healthy controls (*P* < .001). For instance, in the UK Biobank, the average RAG values for healthy participants and patients with type 1 diabetes were 0.12 years and 3.61 years, respectively, averaged over all model types. Similarly, in the BRSET dataset, these values were 0.60 years for healthy participants and 4.25 years for those with type 1 diabetes, averaged across all models.

## Discussion

The key outcome of this work is the demonstration that the newly proposed distributed learning system, employing the 8-bit quantized RETFound-based LRH model, can deliver a computationally highly efficient retinal age prediction performance that is comparable to that of centralized learning models. Additionally, the 8-bit quantized RETFound-based LRH model is more efficient to optimize than larger neural network architectures due to the small number of trainable parameters. It has been shown that the simple linear head model delivers performance comparable to that of more extensive MLP architectures trained using RETFound-based features. Furthermore, when using data from 2400 participants, the TM and FL strategies yielded MAEs of 3.42 years and 3.41 years, respectively. These results surpass the MAE of 3.55 years reported by Zhu et al., who utilized the Xception deep learning architecture for centralized biological retinal age estimation.^1–3^ Such an approach is invaluable, particularly for rural and resource-limited regions, as the conventional model of data centralization not only poses logistical challenges but also raises significant privacy and security concerns. By validating the effectiveness of distributed learning for retinal age prediction, this work provides a promising path forward that could significantly enrich the volume of datasets available for ML training without compromising on privacy or requiring extensive resources, thereby helping to enhance healthcare quality across diverse settings. Moreover, the framework has shown promising clinical utility by illustrating that the RAG values estimated using the trained models have potential to detect type 1 diabetes in 2 different databases.

The adoption of an 8-bit quantized RETFound encoder for feature extraction within the distributed learning framework provides a substantial improvement in terms of memory and processing efficiency. Remarkably, this compression does not significantly compromise the model's predictive accuracy. The performance of the quantized models was shown to closely align with that of their unquantized centralized counterparts, demonstrating only minimal differences in accuracy. Although these experiments were specifically performed for centralized models, we assume that similar results extend to FL and TM models, given that their age prediction capabilities were shown to be comparable to centralized models.

Previous work has demonstrated the feasibility of deploying ViT models on mobile devices such as smartphones.^21–23^ Therefore, by further reducing the memory demands, the developed framework becomes particularly promising for training on devices with limited processing power and memory, such as smartphones, tablets, and other mobile devices that can be used in remote and underserved healthcare settings.^34^ The implications of these findings extend beyond the immediate context of retinal age prediction, suggesting a promising avenue for reducing the memory requirements needed to utilize large transformer models across a broader spectrum of medical image analysis tasks.^35^

A critical observation from our analysis is that both FL and TM methodologies demonstrate comparable efficacy in their predictive capabilities. Yet, an interesting divergence is noted in their operational dynamics, specifically regarding the process of model convergence. More precisely, the TM strategy is distinguished by its requirement for a relatively lower number of local updates on client devices to reach convergence. Also, the TM strategy provided faster convergence performance for smaller datasets than the FL models. This nuanced understanding of the convergence behaviors inherent to FL and TM strategies is important when utilizing distributed learning systems, especially in contexts sensitive to computational resources and data transfer volumes. Specifically, within the context of these experiments, the TM strategy, with its fewer required local updates, emerges as a notably more resource-efficient pathway for model training, especially for smaller training dataset sizes. The value of using the TM strategy for small training dataset sizes is supported by previous research from Souza et al^16^

There are a few limitations and challenges that warrant the need for further exploration in future studies. First, the reliance on data acquired through the standardized protocols of the UK Biobank as the primary data source limits the variability in the dataset. Expanding the dataset to include an increased range of imaging devices and less standardized imaging protocols could benefit the generalizability of the method. Furthermore, since the data originate from a UK-based population, broadening this work to include data from more diverse global sources would be important to assess the applicability of the findings across different populations and demographic settings. This is also highlighted by the evaluation using the BRSET dataset, which showed trends not only consistent with those found in the UK Biobank but also slightly worse mean average errors. This is another clear motivation for increasing data variability through distributed learning. Additionally, the UK Biobank contains a substantial quantity of low-quality fundus images, necessitating the exclusion of many images during quality control. This issue was previously highlighted by MacGillivray et al., who observed that a substantial portion of the retinal images in the UK Biobank are of insufficient quality for automated analysis.^36^ Exclusion of poor-quality images is crucial to ensure that only clean data from healthy subjects is utilized for training. However, such exclusions reduce the dataset’s size and diversity, which may limit the generalizability of models trained on this data. Second, the current work focuses exclusively on the scenario where clients contain fundus images from one subject. Investigating scenarios where clients hold data from multiple subjects could offer additional insights and broaden the participation of clinics and individuals within the framework. Third, although conducting age gap analysis for additional specific disease groups falls outside the scope of the current work, this is an exciting avenue for future research given the promising results for preliminary analysis using patients with type 1 diabetes.

## Conclusion

This work introduces an innovative distributed learning framework that utilizes features extracted from retinal fundus images using an 8-bit quantized RETFound encoder to train a compact LHR model for predicting retinal age. Its high computational and memory efficiency makes this approach particularly well-suited for settings with limited resources, thereby enhancing the ability to include training data from traditionally underrepresented groups. Moreover, by facilitating the inclusion of more data from these groups, this work contributes to the improvement of RAG's accessibility as a key biomarker for worldwide disease screening.

## Supplementary Material

ocae220_Supplementary_Data

## Data Availability

UK Biobank data are available at https://www.ukbiobank.ac.uk/. BRSET data are available at https://physionet.org/content/brazilian-ophthalmological/1.0.0/.

## Computer code

Code for this work is provided at https://github.com/chrisnielsen/foundation-distributed-learning.
