# Supplementary material for: Foundation model-driven distributed learning for enhanced retinal age prediction
Source: J Am Med Inform Assoc. 2024 Sep 3;31(11):2550–9. doi: 10.1093/jamia/ocae220 (PMC11491655; doi:10.1093/jamia/ocae220)
Supplement: ocae220_Supplementary_Data [file ocae220_supplementary_data.zip › ocae220_Supplementary_Data/Supp Table 1.pdf]

Supplementary Table 1. Mean absolute error for the trained models.

Number of simulated clients	Learning strategy	MAE on internal validation dataset	MAE on external testing dataset
150	Centralized	3.88 ± 0.11	3.98 ± 0.10
300	Centralized	3.65 ± 0.09	3.79 ± 0.08
600	Centralized	3.53 ± 0.09	3.66 ± 0.07
1200	Centralized	3.45 ± 0.10	3.57 ± 0.07
2400	Centralized	3.37 ± 0.08	3.50 ± 0.09
150	FL	3.96 ± 0.14	4.11 ± 0.12
300	FL	3.71 ± 0.14	3.92 ± 0.07
600	FL	3.57 ± 0.10	3.75 ± 0.08
1200	FL	3.49 ± 0.10	3.67 ± 0.06
2400	FL	3.41 ± 0.10	3.56 ± 0.10
150	TM	3.89 ± 0.13	4.07 ± 0.13
300	TM	3.68 ± 0.12	3.90 ± 0.09
600	TM	3.57 ± 0.10	3.73 ± 0.09
1200	TM	3.48 ± 0.09	3.67 ± 0.13
2400	TM	3.42 ± 0.09	3.60 ± 0.14
