# Supplementary material for: Foundation model-driven distributed learning for enhanced retinal age prediction
Source: J Am Med Inform Assoc. 2024 Sep 3;31(11):2550–9. doi: 10.1093/jamia/ocae220 (PMC11491655; doi:10.1093/jamia/ocae220)
Supplement: ocae220_Supplementary_Data [file ocae220_supplementary_data.zip › ocae220_Supplementary_Data/Supp Table 2.pdf]

Supplementary Table 2. Average computational and memory transfer overhead per simulated client to train the LRH model using distributed learning.

Number of simulated clients	Learning strategy	Average computation (kFLOPs) per client	Average data transfer (MBs) per client
150	FL	3780.77 ± 740.27	1.89 ± 0.37
300	FL	2849.41 ± 599.33	1.43 ± 0.30
600	FL	2196.23 ± 386.94	1.10 ± 0.19
1200	FL	1698.27 ± 321.67	0.85 ± 0.16
2400	FL	1389.36 ± 249.11	0.70 ± 0.12
150	TM	436.48 ± 82.99	0.22 ± 0.04
300	TM	456.46 ± 50.74	0.23 ± 0.03
600	TM	470.29 ± 67.27	0.24 ± 0.03
1200	TM	531.77 ± 67.65	0.27 ± 0.03
2400	TM	540.99 ± 194.51	0.27 ± 0.10
