# Supplementary material for: Foundation model-driven distributed learning for enhanced retinal age prediction
Source: J Am Med Inform Assoc. 2024 Sep 3;31(11):2550–9. doi: 10.1093/jamia/ocae220 (PMC11491655; doi:10.1093/jamia/ocae220)
Supplement: ocae220_Supplementary_Data [file ocae220_supplementary_data.zip › ocae220_Supplementary_Data/Supp Table 3.pdf]

Supplementary Table 3. ICD-10 codes used to identify patients with type 1 diabetes.

Disease group	ICD-10 codes
Type 1 diabetes	E08-E08.11, E08.3-E08.9, E10-E10.11, E10.3-E11.1, E11.3-E12.1, E12.3-E13.11, E13.3-E14.1, E14.3-E14.9, R73-R73.9, Z13.1, Z83.3
