# Supplementary material for: Foundation model-driven distributed learning for enhanced retinal age prediction
Source: J Am Med Inform Assoc. 2024 Sep 3;31(11):2550–9. doi: 10.1093/jamia/ocae220 (PMC11491655; doi:10.1093/jamia/ocae220)
Supplement: ocae220_Supplementary_Data [file ocae220_supplementary_data.zip › ocae220_Supplementary_Data/Supp Table 4.pdf]

Supplementary Table 4. Average RAG values for healthy participants and participants with type 1 diabetes.

Dataset	Learning strategy	Average RAG for healthy group	Average RAG for diabetes group
UK Biobank	Centralized	0.11 ± 0.08	3.63 ± 0.32
UK Biobank	FL	0.13 ± 0.10	3.60 ± 0.34
UK Biobank	TM	0.12 ± 0.09	3.61 ± 0.31
BRSET	Centralized	0.58 ± 0.17	4.28 ± 0.38
BRSET	FL	0.62 ± 0.16	4.24 ± 0.35
BRSET	TM	0.60 ± 0.18	4.23 ± 0.36
